# Exploring sphingolipid metabolism-related biomarkers for Parkinson’s disease: a transcriptomic analysis

**DOI:** 10.3389/fneur.2025.1548322

**Published:** 2025-06-04

**Authors:** Guohong Wang, Xingnan Zhou

**Affiliations:** ^1^Department of Neurology, The Third Affiliated Hospital of Anhui Medical University (the First People's Hospital of Hefei), Hefei, China; ^2^Department of Neurology, School of Clinical Medicine, Bengbu Medical University, Bengbu, China

**Keywords:** Parkinson’s disease, sphingolipid metabolism, biomarkers, function, immune, regulation

## Abstract

**Background:**

Related studies have pointed out that sphingolipids and their metabolites are involved in the growth of neurons, and were associated with the occurrence and development of central nervous system diseases. However, the role of sphingolipid metabolism-related genes (SMRGs) in Parkinson’s Disease (PD) have not been fully elucidated.

**Methods:**

In this study, PD-related transcriptome data were extracted from the Gene Expression Omnibus (GEO) database. The DE-SMRGs were obtained by intersecting the differentially expressed genes (DEGs) screened by “limma” and the SMRGs, and the functional enrichment analysis of these DE-SMRGs was conducted by “clusterProfiler.” Then, the biomarkers of PD were screened by protein–protein interaction (PPI) analysis. Based on this, three methods, including functional similarity analysis, co-expression analysis, and gene set enrichment analysis (GSEA) were conducted to study the functions of biomarkers. Moreover, the immune cell infiltration analysis was used to further study the immune-related mechanisms of biomarkers in PD. Furthermore, the mRNA-miRNA regulatory network was constructed to reveal the potential regulation of biomarkers. Finally, the targeted drugs of biomarkers were predicted for the clinical treatment of PD.

**Results:**

A totals of 14 DE-SMRGs were obtained by intersecting 1,139 DEGs and 97 SMRGs, and these genes were involved in the ceramide metabolic process. Five biomarkers, including Arylsulfatase B (ARSB), N-Acylsphingosine Amidohydrolase 1 (ASAH1), Galactosidase Beta 1 (GLB1), Hexosaminidase Subunit Beta (HEXB), and Prosaposin (PSAP) were screened, and they played an important role in the immune process and are associated with immune cells such as macrophages. The expression of biomarkers was validated in clinical human samples by quantitative reverse transcription polymerase chain reaction (qRT-PCR). The expression levels of GLB1, ASAH1 and PSAP were increased in human samples, which were consistent with the bioinformatics analysis results. Moreover, the mRNA-miRNA regulatory network was constructed, and it was worth noting that hsa-miR-134-5p could regulate ARSB and ASAH1, hsa-miR-27a-3p and hsa-miR-27b-3p could regulate ASAH1 and PSAP at the same time. In addition, Chondroitin sulfate could target ARSB and HEXB simultaneously.

**Conclusion:**

This study identified five sphingolipid metabolism-related biomarkers (ARSB, ASAH1, GLB1, HEXB, and PSAP) of PD. This finding provided the possibility of SMRGs as biomarkers for PD.

## 1 Introduction

Parkinson’s Disease (PD) is the second most common degenerative disease of the central nervous system, showing an increase in incidence and prevalence with advancing age (1). Its characteristics are the selective loss of dopamine neurons in the substantia nigra striatum of the midbrain, as well as the formation of Lewy bodies, which are mainly formed by the aggregation of misfolded alpha synuclein (2). So far, the pathogenesis of PD remains unclear and may be related to genetic factors, environmental toxins and so on (3, 4). As the pathogenesis of PD is still unclear, there is still a lack of reliable and easy diagnostic methods. Previous studies have explored various types of potential PD biomarkers, covering multiple aspects such as clinical, pathological, biochemical, and genetic aspects (5–7). However, these biomarkers are either limited due to their inability to be applied in the diagnosis of the early stages of the disease or have not been fully validated to ensure their effectiveness (8).

Notably, considering a combination of multiple biomarkers from different levels may maximize their utility and thus improve the accuracy of PD early—stage diagnosis (9). Therefore, it is particularly important to continuously explore and develop new biomarkers with high diagnostic sensitivity and specificity. In recent years, an increasing number of studies have discovered a close association between sphingolipid metabolism and PD. Galper et al. found that the lipid changes in PD patients mainly involve the sphingolipid and phospholipid metabolic pathways, as well as the oxidative phosphorylation/thermogenesis and insulin resistance pathways. These lipid changes can distinguish PD patients from healthy controls, and alterations in serum lipid species may occur in the early stages of prodromal PD (10). As a major class of lipids, sphingolipids mainly include sphingosine-1-phosphate (S1P), ceramide, sphingomyelin (SM), and various glycosphingolipids (GSLs). They are crucial components of the biological membrane structure and can maintain the barrier function and fluidity of the cell membrane (11). Research has shown that the accumulation of GSLs can disrupt the normal degradation function of lysosomes, thereby affecting fundamental life processes such as mitochondrial respiration and autophagy. Meanwhile, lysosomal damage triggers the accumulation of *α*-synuclein, which in turn exacerbates lysosomal dysfunction (12). Karim et al. also found that glycosphingolipids can influence neuroinflammation in PD through mechanisms such as activating inflammasomes, promoting the secretion of pro-inflammatory cytokines, altering calcium homeostasis, affecting the permeability of the blood–brain barrier, recruiting peripheral immune cells, or generating autoantibodies (13). Sphingolipids are mainly synthesized through two major pathways, including the *de novo* synthesis pathway and the salvage pathway. The de novo synthesis pathway, the primary route for sphingolipid production, mainly occurs in the endoplasmic reticulum (ER). In this pathway, serine and palmitoyl-CoA serve as the starting substrates, and through a series of enzymatic reactions, sphingosine and ceramide are produced, which are then used to synthesize complex sphingolipid molecules (14). The salvage pathway is the second pathway of sphingolipid metabolism. In this pathway, sphingosine is salvaged through re-acylation, resulting in the generation of ceramide or its derivatives, and this pathway plays an important role in ceramide metabolism and function (15). Increasing evidence indicates that impaired ceramide metabolism is associated with PD, and inhibiting ceramide synthesis can reduce *α*-synuclein lesions in PD cell models (16). However, the biological role of sphingolipid metabolism-related genes (SMRGs) in PD is limited.

In this study, we screened and identified SMRG in PD using bioinformatics methods, and validated the expression of SMRG in clinical human samples using quantitative reverse transcription polymerase chain reaction (qRT-PCR). Although previous studies have clearly identified the importance of sphingolipids in Parkinson’s disease, this study extends the previous research by combining genomic, proteomic, and metabolomic data to more comprehensively explore the mechanism of sphingolipids in Parkinson’s disease, providing an important theoretical basis for further in-depth research. A flow chart for this study is shown in Supplementary Figure 1B.

## 2 Materials and methods

### 2.1 Data extraction

The PD-related datasets were downloaded from the GEO database. Peripheral blood samples were chosen because it is more convenient to obtained blood samples than to take brain tissue directly, and could reflect the overall physiological and pathological state of the body and may be associated with neuroinflammation and immune responses in the brain. The GSE100054 dataset contains 10 PD and nine healthy control (HC) peripheral blood samples, and the GSE99039 dataset contains 205 PD and 233 HC whole blood samples. Besides, a totals of 97 SMRGs were obtained from the previous studies.

### 2.2 Functional enrichment analysis of differentially expressed SMRGs (DE-SMRGs)

Differential expression analysis aimed to identify genes that were differentially expressed between different sample groups. The differentially expressed genes (DEGs) between 10 PD and nine HC samples in the GSE100054 dataset were compared by the “limma” R package (version 3.52.4) (|log2FC| ≥ 0.5, adj. *p*-value < 0.05). Then, the DE-SMRGs were obtained by intersecting the DEGs and SMRGs. Next, the functional enrichment analysis of these DE-SMRGs was conducted by the “clusterProfiler” R package (version 4.7.1) (adj. *p*-value < 0.05).

### 2.3 Screening for the biomarkers of PD

In order to identified the PD biomarkers with high diagnostic accuracy and potential clinical applications, we performed protein–protein interaction (PPI) network, model validation and expression validation analyses. Firstly, the PPI network of DE-SMRGs was constructed by “STRING” (Confidence = 0.4, minimum required interaction score = 0.4, species = 9,606) (version 11.5), construct human protein interaction data that removed self-interactions and contained only multiple types of protein interactions with scores of 400 or more, which were and the hub genes (Top genes) were selected by “Closeness,” and further defined as the biomarkers of PD for the following analyses. Then, the receiver operating characteristic (ROC) curves of each biomarker were drawn to study the ability to distinguish the PD and HC by the “pROC” R package (version 1.18.0). In addition, the expressions of these biomarkers between PD and HC were compared in both the GSE100054 and GSE99039 datasets, respectively.

### 2.4 Function analyses of biomarkers

In this study, three methods were used to further explore the functions of biomarkers. Firstly, the correlations among these biomarkers were calculated by the “psych” R package (version 2.2.9), and the functional similarity analysis was analyzed by the “GOSemSim” R package (version 2.24.0). Secondly, the co-expression network of biomarkers and top 20 genes with the highest correlation with biomarkers was constructed in the GeneMANIA database. Thirdly, the correlation coefficients between each biomarker and all genes in GSE100054 were calculated, and GSEA was performed to study the Kyoto Encyclopedia of Genes and Genomes (KEGG) pathways of each biomarker by “clusterProfiler” R package (version 4.7.1) (adj. *p*-value < 0.05).

### 2.5 Immune infiltration analysis

We wished to reveal possible differences in immune cell composition and biomarker-immune cell correlations between PD patients and healthy controls (HC). Thus, the proportions of 28 immune cells between PD and HC samples in GSE100054 were calculated by the “single sample gene set enrichment analysis (ssGSEA)” algorithm and compared by the “Wilcoxon” test. Moreover, the correlations between biomarkers and differential immune cells were further studied by “Spearman”.

### 2.6 The potential regulatory mechanism analysis of biomarkers

To study the potential regulatory mechanism analysis of biomarkers, the targeted miRNAs of biomarkers were predicted in the Starbase database, and the mRNA-miRNA regulatory network was constructed by “Cytoscape” (version 3.9.1).

### 2.7 Drug prediction

Furthermore, the targeted drugs of biomarkers were predicted in the DrugBank database for clinic treatment of PD, and the gene-drug network was also constructed by “Cytoscape” (version 3.9.1).

### 2.8 Clinical validation of biomarkers by quantitative real-time reverse transcription polymerase chain reaction (qRT-PCR) analysis

To further explore the role of biomarkers in PD, biomarker expression was confirmed by qRT-PCR. During this experiment, a total of 10 clinical blood samples, comprising five control and five disease samples, were acquired from normal and PD patients in the Binhu Branch of the First People’s Hospital of Hefei. The research was approved by the Ethical Committee of the First People’s Hospital in Hefei (Ethics number: 2024-05) and conducted in accordance with the Declaration of Helsinki (as revised in 2013). All participants in the present study completed and submitted informed consent.

Total RNA was prepared from blood using PBMC isolate and TRIZol reagent. Reverse transcription was performed using the SureScript First-Strand cDNA Synthesis Kit to obtain cDNAs. qRT-PCR was performed as follows: a total of 40 cycles, 95°C for 1 min, 95°C for 20s, 55°C for 20s, and 72°C for 30s. GAPDH was used as the internal reference genes. The qRT-PCR primers were listed in Table 1. The relative expression levels of prognosis genes were calculated by the 2^-△△^ct method.

**Table 1:** qRT-PCR primers.

Primer	Primer sequence
GLB1 F	GGCCCACAACTCATCCAACT
GLB1 R	AAACTGCTGCCATCGCCTT
HEXB F	CACCGTGATCTCCGCACC
HEXB R	CGTGACCGTCGCTAGAACTC
ASAH1 F	GAGCTACTCGCGTCCGCC
ASAH1 F	AACAGCAGGGAAGACAGTTGG
PSAP R	ATGCAAAGACGTTGTCACCG
PSAP R	GGGAGGTAGGAGTCCACTATCT
ARSB F	AATGGCACAAAGCCTCTGGA
ARSB R	CACTCAGGCCAGTACGGTG
β-actin-GAPDH F	CGAAGGTGGAGTCAACGGATTT
β-actin-GAPDH R	ATGGGTGGAATCATATTGGAAC

### 2.9 Statistical analysis

All analyses were conducted using the R language. Differences between the two groups were compared by “Wilcoxon” test. The experimental part of the analysis of the experimental result data of qRT-PCR was evaluated using t-test. If not specified above, *p* < 0.05 was regarded as statistically significant.

## 3 Results

### 3.1 DE-SMRGs were involved in the ceramide metabolic process

**Figure 1:**
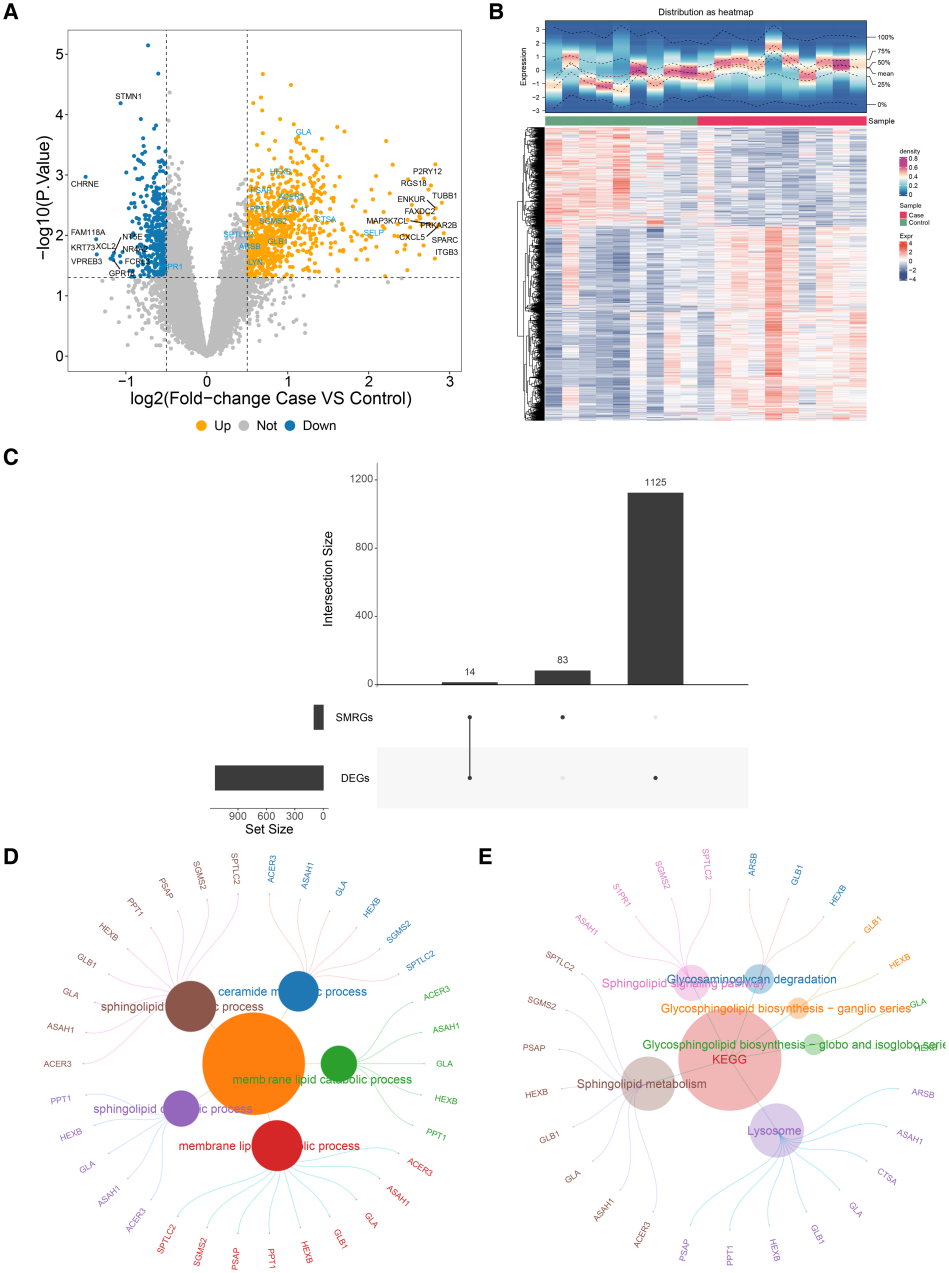
Screening of differentially expressed genes and their functional analysis. **(A)** The left panel shows the vmap drawn by differential analysis. Orange represents up-regulated genes and blue represents down-regulated genes. **(B)** The right panel shows a heat map of differential gene expression. Red represents up-regulated genes and blue represents down-regulated genes. The horizontal coordinate direction is for samples and the vertical coordinate direction is for differentially expressed genes. **(C)** Differentially expressed genes and SMRGs take intersections to obtain a Venn diagram of DENRGs. **(D)** The Results of GO enrichment analysis. Display the TOP5 pathway (significance ranking) and its contained genes, with the size of the circle representing the number of contained genes. **(E)** The results of KEGG enrichment analysis. Display the TOP5 pathway (significance ranking) and its contained genes, with the size of the circle representing the number of contained genes.

By differential expression analysis, there were 1,139 DEGs (754 up-regulated and 385 down-regulated) between PD and HC samples in the GSE100054 dataset (Figures 1A,B). Then, 14 DE-SMRGs were obtained by intersecting 1,139 DEGs and 97 SMRGs (Table 2), which included Alkaline Ceramidase 3 (ACER3), Arylsulfatase B (ARSB), N-Acylsphingosine Amidohydrolase 1 (ASAH1), Cathepsin A (CTSA), Galactosidase Alpha (GLA), Galactosidase Beta 1 (GLB1), Hexosaminidase Subunit Beta (HEXB), v-yes-1 Yamaguchi sarcoma viral related oncogene homolog (LYN), Palmitoyl-Protein Thioesterase 1(PPT1), Prosaposin (PSAP), Selectin P (SELP), Sphingosine-1-Phosphate Receptor 1 (S1PR1), Sphingosine-1-Phosphate Receptor 1 (SGMS2), and Serine Palmitoyltransferase Long Chain Base Subunit 2 (SPTLC2) (Figure 1C).

**Table 2:** Information on the differential gene DE-SMRGs.

GLA
HEXB
PSAP
ACER3
PPT1
CTSA
ASAH1
SGMS2
SPTLC2
GLB1
ARSB
SELP
S1PR1
LYN

From the perspective of functions, these 14 DE-SMRGs were enriched to sphingolipid metabolic.

(biosynthetic and catabolic) process, sphingosine metabolic process, membrane lipid metabolic process, ceramide metabolic process, and etc. 99 Gene Ontology (GO) functions (Figure 1D). And these genes were associated with sphingolipid metabolism, glycosaminoglycan degradation, sphingolipid signaling pathway, glycosphingolipid biosynthesis, galactose metabolism, and etc. eight KEGG pathways (Figure 1E).

### 3.2 ARSB, ASAH1, GLB1, HEXB, and PSAP were screened as biomarkers of PD

**Figure 2:**
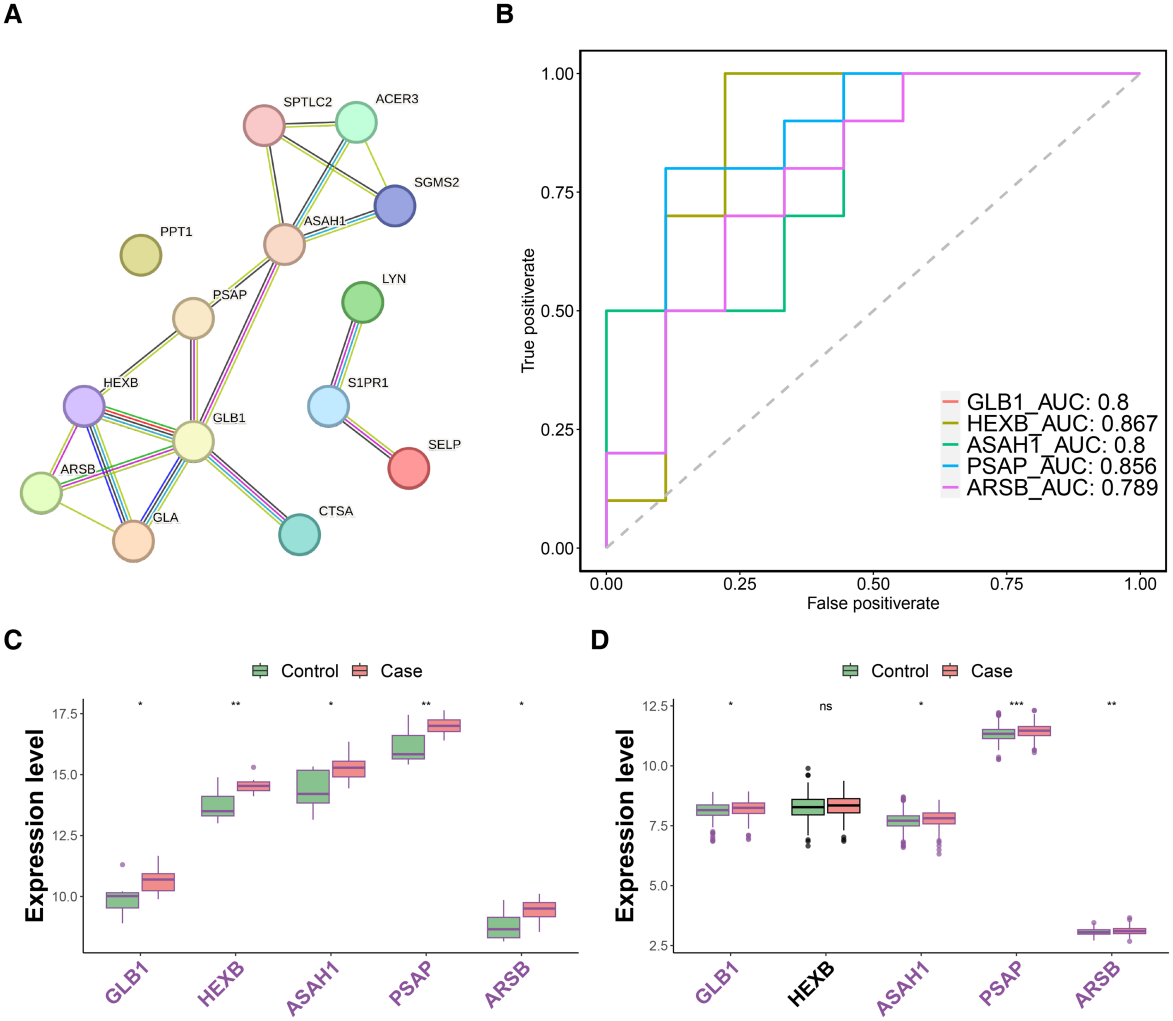
Screening of biomarkers and validation of their expression. **(A)** The plot of PPI network. **(B)** The receiver operating characteristic curve (ROC curve) of five biomarkers (ARSB, ASAH1, GLB1, HEXB, and PSAP). Intergroup expression of biomarkers in both GSE100054 (Left) **(C)** and GSE99039 (Right) **(D)** datasets. Purple represents upregulation, green represents downregulation, and black represents insignificant. ns represents *p* > 0.05, * represents *p* ≤ 0.05, ** represents *p* ≤ 0.01, *** represents *p* ≤ 0.001.

Furthermore, the PPI network of the above 14 DE-SMRGs was constructed with 14 nodes and 19 protein interaction relationship pairs, and five biomarkers (Top5), including Arylsulfatase B (ARSB), N-Acylsphingosine Amidohydrolase 1 (ASAH1), Galactosidase Beta 1 (GLB1), Hexosaminidase Subunit Beta (HEXB), and Prosaposin (PSAP) were selected (Figure 2A; Table 3). The area under the curve (AUC) values of all these five biomarkers were greater than 0.7 (Figure 2B). Except for HEXB, the expression trends of other genes in the GSE100054 and GSE99039 datasets are consistent, and these genes are highly expressed in the PD group (Figures 2C,D).

**Table 3:** Biomarker enrichment results.

node_name	MCC	DMNC	MNC	Degree	EPC	Bottleneck	Eccentricity	Closeness	Radiality	Betweenness	Stress	Clusteringcoefficient
PPT1	0	0	0	0	1	0	0	0	0	0	0	0
SELP	1	0	1	1	1.633	1	0.10714	1.5	0.64286	0	0	0
S1PR1	2	0	1	2	1.869	3	0.21429	2	0.75	2	2	0
LYN	1	0	1	1	1.616	1	0.10714	1.5	0.64286	0	0	0
CTSA	1	0	1	1	3.76	1	0.2381	4.5	1.5873	0	0	0
PSAP	4	0.30898	3	3	5.366	1	0.35714	6	1.98413	4	8	0.66667
GLB1	11	0.32413	5	6	6.034	10	0.35714	7.5	2.22222	38	44	0.33333
HEXB	8	0.37893	4	4	5.482	1	0.2381	6	1.8254	2	4	0.66667
GLA	6	0.46346	3	3	5.148	1	0.2381	5.5	1.74603	0	0	1
ARSB	6	0.46346	3	3	5.181	1	0.2381	5.5	1.74603	0	0	1
SGMS2	6	0.46346	3	3	4.81	1	0.2381	5.33333	1.66667	0	0	1
ASAH1	8	0.46346	3	5	5.743	10	0.35714	7	2.14286	36	42	0.4
SPTLC2	6	0.46346	3	3	4.862	1	0.2381	5.33333	1.66667	0	0	1
ACER3	6	0.46346	3	3	4.734	1	0.2381	5.33333	1.66667	0	0	1

### 3.3 Functional analysis of biomarkers

**Figure 3:**
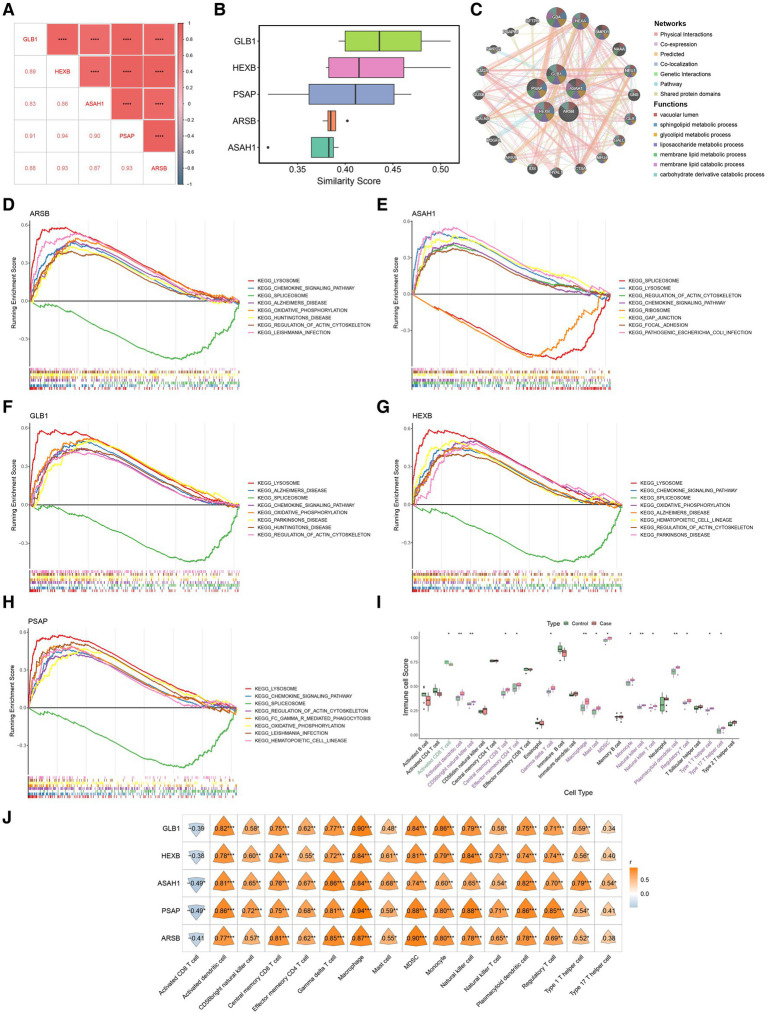
Functional and immune infiltration studies of biomarkers. **(A)** Correlations of biomarkers. The lower triangle represents the correlation coefficient, and the upper triangle represents the significance of the correlation. **(B)** Functional similarity analysis among these five biomarkers. **(C)** Gene gene interactions for constructing biomarkers Network (GGI). **(D–H)** Results of GSEA analysis of biomarker. **(I)** Immune cells scores analysis of these five biomarkers in Case and control group. Purple represents Case>Control, green represents Case<Control, black indicates insignificant. The top represents significance, where ns represents *p* > 0.05, * represents *p* ≤ 0.05, ** represents *p* ≤ 0.01, and *** represents *p* ≤ 0.001, **** indicates *p* ≤ 0.0001. **(J)** Correlation between biomarkers and immune cells.

The one-by-one correlations among these five biomarkers were higher than 0.8 (Figure 3A). The functional similarity analysis results showed that GLB1 has higher functional similarity scores and might be the most important gene, and GLB1, HEXB, and PSAP have similar functions (Figure 3B). The co-expression network of these five biomarkers and top20 genes with the highest correlation with biomarkers was constructed. Notably, most of these biomarkers were associated with vacuolar lumen, sphingolipid metabolic process, glycolipid metabolic process, liposaccharide metabolic process, and membrane lipid metabolic process. Besides, Glucosylceramidase Beta (GBA), Hexosaminidase Subunit Alpha (HEXA), Sphingomyelin Phosphodiesterase 1 (SMPD1), Neuraminidase 1 (NEU1), Galactosidase Alpha (GLA), and etc. were also associated with these functions (Figure 3C). The five biomarkers were enriched as follows, GLB1 was predominantly enriched in the lysosomal pathway, which was related to Parkinson’s disease, as well as in the pathways associated with sphingolipid metabolism and Alzheimer’s disease. ARSB was mainly enriched in the lysosome, chemokine signaling pathway, spliceosome, Alzheimer’s disease, oxidative phosphorylation, Huntington’s disease, regulation of actin cytoskeleton, and Leishmania infection pathways. ASAH1 was primarily enriched in the spliceosome pathway and also in the lysosome, regulation of actin cytoskeleton, chemokine signaling pathway, ribosome, junctional focal adhesion, and pathogenic *Escherichia coli* infection pathways. HEXB was mainly enriched in the lysosome, chemokine signaling pathway, spliceosome, oxidative phosphorylation, Alzheimer’s disease, hematopoietic cell lineage, regulation of actin cytoskeleton, and Parkinson’s disease pathways. PSAP was predominantly enriched in the lysosome, chemokine signaling pathway, spliceosome, oxidative phosphorylation, alzheimer’s disease, regulation of actin cytoskeleton, FC gamma R-mediated phagocytosis, oxidative phosphorylation, Leishmania infection, hematopoietic cell lineage, and other pathways (Figures 3D–H).

### 3.4 Biomarkers significantly correlate with immune function

Biomarkers were implicated in immune-related pathways, that activated dendritic cell, CD56dim natural killer cell, central memory CD8 T cell, macrophage, and etc. 15 immune cell were significantly increased, and only the activated CD8 T cell was significantly decreased in PD group (Figure 3I). To revealled the correlation between biomarkers and immune cells, correlations between biomarkers and immune cell enrichment scores for between-group differences were analyzed using Spearson correlation. Among them, GLB1 and PSAP had the highest positive correlations with macrophage (r = 0.90, r = 0.94), ARSB had the highest positive correlations with myeloid derived suppressor cell (MDSC) (r = 0.90), ASAH1 had the highest positive correlations with gamma delta T cell (r = 0.86), and HEXB had the highest positive correlations with natural killer cell and macrophage (r = 0.84) (Figure 3J).

### 3.5 The regulation mechanism analysis of biomarkers

**Figure 4:**
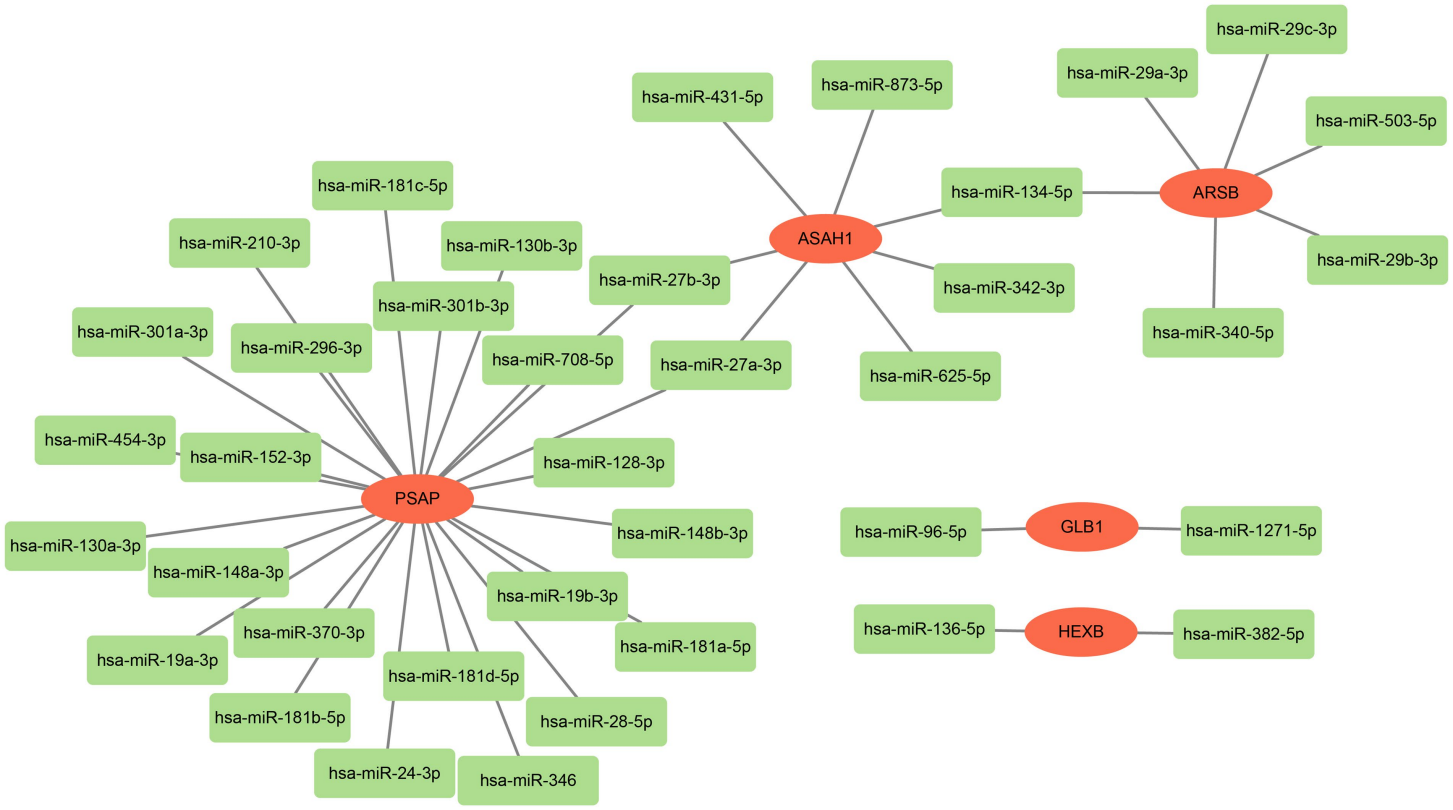
The regulation mechanism analysis of biomarkers. The red pattern represents biomarkers, and the green represents miRNAs.

The mRNA-miRNA regulatory network was constructed with five biomarkers and 38 miRNAs. Among them, there were 24 targeted miRNAs of PSAP, six targeted miRNAs of ARSB, seven targeted miRNAs of ASAH1, and two targeted miRNAs of GLB1/HEXB. Moreover, it was worth noting that hsa-miR-134-5p was the common targeted miRNA of ARSB and ASAH1, and there were two common targeted miRNAs (hsa-miR-27a-3p and hsa-miR-27b-3p) between ASAH1 and PSAP (Figure 4).

### 3.6 Drug prediction

**Figure 5:**
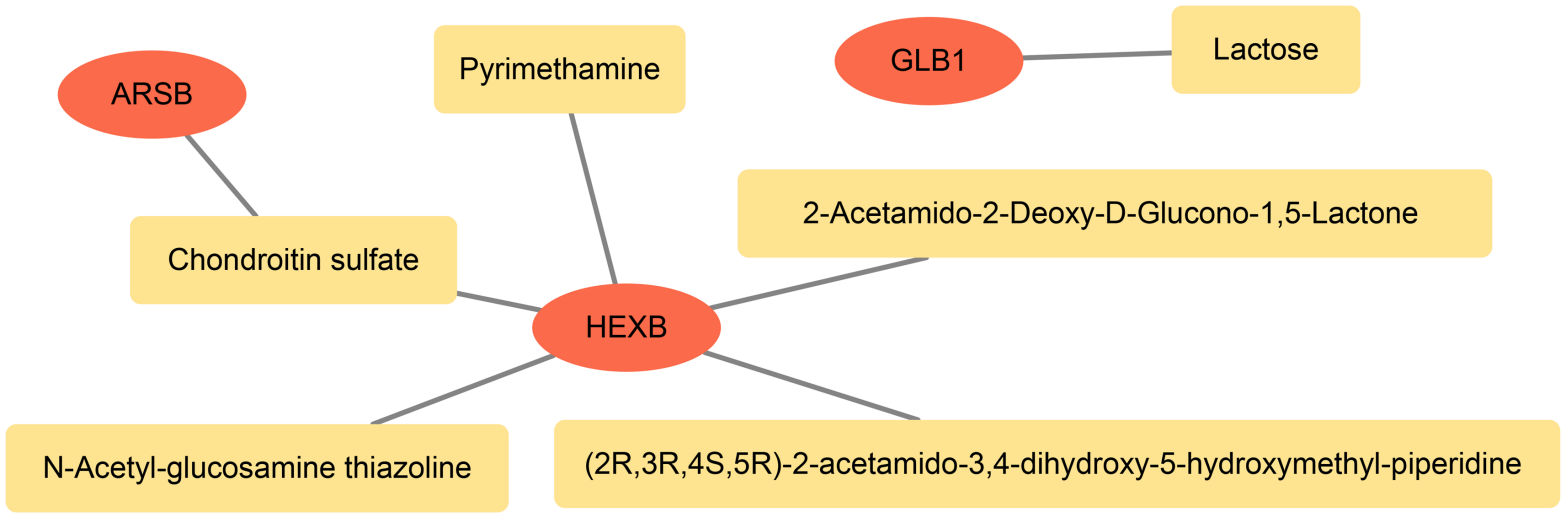
The relationship network between genes and drugs. The red pattern represent biomarkers and the orange represents drugs.

Furthermore, totals of six targeted drugs were predicted, and among them, Chondroitin sulfate was the targeted drug of ARSB, Lactose was the targeted drug of GLB1, and there were five targeted drugs of HEXB. It was worth noting that Chondroitin sulfate could target ARSB and HEXB simultaneously (Figure 5).

### 3.7 Biomarkers were confirmed by RT-qPCR

**Figure 6:**
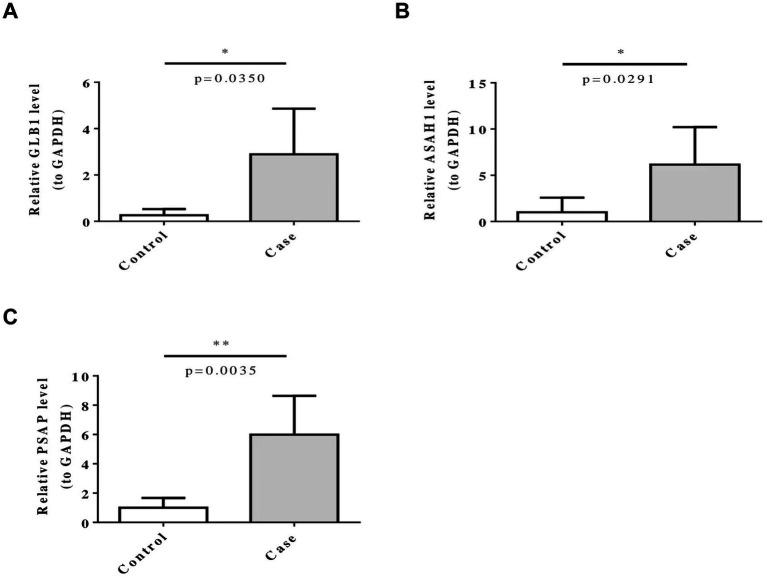
Five biomarkers (ARSB, ASAH1, GLB1, HEXB, and PSAP) were confirmed by RT-qPCR. ns represents *p* > 0.05, * represents *p* ≤ 0.05, ** represents *p* ≤ 0.01.

The RT-qPCR results showed that the expression levels of GLB1, ASAH1 and PSAP in the PD group were significantly higher compared with the control group (Figure 6).

## 4 Discussion

Currently, the pathogenesis of Parkinson’s Disease (PD) remains obscureand may be related to genetic factors, autoimmunity and so on (3, 4). Recent studies have found that the abnormalities of some genes may lead to the hypersensitivity of PD patients to environmental toxins, inflammation and other damages, which may be a risk factor for PD (17). In addition, sphingolipid metabolism plays an important role in PD (18). Nevertheless, there is limited research on how SMRGs play a part in PD, it is worth exploring in depth. In this study, five SMRGs [including Arylsulfatase B (ARSB), N-Acylsphingosine Amidohydrolase 1 (ASAH1), Galactosidase Beta 1 (GLB1), Hexosaminidase Subunit Beta (HEXB), and Prosaposin (PSAP)] were selected as biomarkers for PD. These five biomarkers are all correlated with GBA. Research has shown that GBA gene mutations are the most important genetic risk factor for Parkinson’s disease (19). This provides a certain basis for us to further study the role of these biomarkers in Parkinson’s disease.

Some studies have shown that GLB1 is an encoding *β*-galactose Hydrolase of Galactosidase, which can participate in the sphingosine, ceramide, galactose, sphingomyelin metabolism (20). When the GLB1 biallele is mutated, β-galactose is deficient, which leads to the deposition of GM1-type gangliosides and affects neuronal function and PD (20, 21). Recent studies have suggested that GLB1 may affect neuronal function through endoplasmic reticulum calcium signaling pathway, NRT1.1-NLP7 pathway, autophagy and inflammasome activation (20). HEXB is the B form of β-cyclohexosidase, which is responsible for the transformation of GM2-type glycosylsphingolipid gangliosides. When HEXB is mutated, it may lead to the deposition of GM2-type gangliosides and have an adverse neurological effect (20). Moreover, it has been found that the specific expression of HEXB in microglia can regulate the immunity of the central nervous system, participate in the TLR4/NF-κB signaling pathway and chronically activate Toll-like receptors (TLRs), influence the progression of PD (22, 23). PSAP is a lysosomal protein with neuroprotective effect, which is involved in the degradation of glycophospholipids. When the mutation of PSAP occurs, the lipid homeostasis of dopamine neurons in PD is unbalanced, which can generate reactive oxygen, lead to lipid oxidation, cause ferroptosis of neurons, and seriously affect the motor symptoms of PD patients (24, 25). It has also been shown that PSAP was highly expressed in macrophages and may participate in Ribosomal protein S6 kinase beta-1 (S6K1) signaling by regulating the phosphorylation of the RPS6-encoded ribosomal protein S6, which regulated the metabolic reprogramming of macrophages and their mediated inflammatory responses, thereby affecting the progress of PD. After knocking down PSAP, both glycolysis and oxidative phosphorylation were significantly down-regulated (26). ASAH1, an acidic ceramidase located in lysosomes, is involved in the metabolism of sphingosine, ceramide and galactose (11). When it is mutated, it will cause calcium accumulation and lead to PD (27). It has been shown that ASAH1 can mediate the degradation of Ceramide, suppress the activation of inflammatory corpuscle NLR Family Pyrin Domain Containing 3 (NLRP3) and downstream events, thus inhibited the occurrence of inflammation in PD (20, 28). ARSB is a gene encoding lysosomal enzymes (arylsulfatase B) required for the degradation of glycosaminoglycans (GAGs), including N-acetyl-d-galactosamine, dermatan sulfate, and chondroitin sulfate. The ARSB mutation, which causes protein defects is known to be the cause of lysosomal storage disease (known as mucopolysaccharidosis type VI) (29). Meanwhile, Blokhin et al. also found an increased expression of ASAH1 in a mouse model of PD, suggesting that it may serve as a biomarker for diagnosing PD (30).

This study revealed that the five biomarkers, GLB1, ARSB, ASAH1, HEXB, and PSAP are all enriched in the lysosomal, chemokine, oxidative phosphorylation, and neurodegenerative disease pathways. Functional defects of lysosomal enzymes can impede the effective clearance of harmful proteins in the nervous system, leading to the selective degeneration of dopaminergic neurons. Therefore, regulatory strategies targeting the lysosomal pathway and its specific components are regarded as a promising approach for exploring new treatment methods for PD (31). Impaired expression of these five biomarkers may affect lysosomal accumulation, cause changes in ceramide levels, and influence the interaction between *α*-synuclein and the cell membrane (32). Another study indicated that viral infections can trigger the production of high levels of cytokines and chemokines in the body. These molecules can cross the blood–brain barrier, leading to microglial activation and inflammation, and ultimately resulting in neuronal cell death (33). Oxidative phosphorylation (OxPhos) is the main pathway for energy production, and many genes associated with PD are related to oxidative phosphorylation, especially Complex I. When it fails, the formation of ATP in these neurons is reduced, thereby inducing apoptosis (34). Notably, the GLB1 and HEXB genes have also been directly linked to the biological pathways associated with PD. These findings further reinforce their status as potential factors influencing the progression of PD. More importantly, GLB1 and HEXB are directly enriched pathways related to Parkinson’s disease, and the above research results fully demonstrates that they are potential factors affecting the progression of Parkinson’s disease.

The results of immune infiltration analysis showed that HEXB, GLB1 and PSAP were associated with macrophages. Numerous studies have shown that microglia cells, which were the most important macrophages in the central nervous system, with the highest numbers, and played a crucial role in immunity in PD (35, 36). Microglia in PD may have two cell subtypes, M1 type promoted inflammation, had neurotoxic effects and M2 type inhibited inflammation, had neuroprotective roles (22, 37). Under normal physiological conditions, microglia were in relative resting state and monitor brain microenvironment by secreting neurotrophic factors to the corresponding neurons to protect the stability of the intracerebral environment (38). However, when *α*-synuclein was abnormally aggregated in PD, it can mediate microglia into a neurotoxic state through TLR2, TLR4-NK-κB and lc3 related phagocytosis. Impaired autophagy of microglia can also lead to increased activity of NLRP3 (NOD-like receptor family, pyrin-containing structural domain of 3) activity in cells, promoting the transition of microglia to a neurotoxic state, thereby increasing phagocytosis and production of proinflammatory cytokines, leading to the death of dopaminergic neurons (39, 40). In addition to causing direct neuron damage, neurotoxic microglia can also amplify PD inflammation within the brain by altering the state of astrocytes (40, 41). Combining the results of functional correlation analysis, the above studies further supported that these five biomarkers may be potential target genes for PD.

As others have pointed out that miRNAs can individually affect multiple strands of mRNAs, altering the gene expression profiles and pathways of target genes, thereby affecting a wide range of cellular functions by altering the protein–protein interaction, and ultimately affecting the etiology and severity of diseases (42). We found that hsa-miR-27a-3p and hsa-miR-27b-3p were common target genes of PSAP and ASAH1, while has-miR-134-5P was a common target gene of ASAH1 and ARSB. Therefore, these miRNAs may play important roles in PD through the lncRNA-miRNA-mRNA regulatory mechanism. By downregulating the expression of FANCD2 and CD44, inhibiting U87 cell and U251 cell proliferation, migration, invasion, hsa-miR-27a-3p can promote cell apoptosis and death (43, 44). It can also participate in the MAPK signaling pathway, PAR signaling pathway, lipid metabolism by acting on the SP3 gene and promote the transformation into M2 type macrophages by restraining the expression of Enhancer of Zeste Homolog 1 (EZH1), thereby inhibiting immune system and central nervous system repair (45, 46). Hsa-miR-27b-3p can down-regulate the expression of Forkhead Box O1 (FOXO1) mRNA, blunt the activation of Akt /FOXO1 pathway, and reduce mitochondrial oxidation and inflammatory response (47). Has-miR-134-5p can involve in immune response, inflammatory response, lipid metabolism and degradation by regulating Interferon Regulatory Factor 1 (IRF1), Nuclear Factor Of Kappa Light Polypeptide Gene Enhancer In B-Cells Inhibitor, Alpha (NFKBIA), and YrdC N6-Threonylcarbamoyltransferase Domain Containing (YRDC) (48). Therefore, we hypothesized that hsa-miR-27a-3p and hsa-miR-27b-3p may participate in the MAPK signaling pathway, NOD-like receptor signaling pathway, lipid metabolism and other processes by regulating PSAP and ASAH1, which would inhibit immune and inflammatory responses and reducing neuronal apoptosis. And hsa-miR-134-5p may also participate in NOD-like receptor signaling pathway and inhibit inflammation activation by regulating ARSB and ASAH1, thereby alleviating PD progression and shortening disease duration.

Subsequently, we predicted targeted drugs for PD. And we found that chondroitin sulfate could target both ARSB and HEXB. Chondroitin sulfate was a kind of sulfated glycosaminoglycan that was widely used in cardiovascular, cerebrovascular and orthopedic diseases, but has not been used in the treatment of PD. Consistent with our findings, recent studies have shown that chondroitin sulfate protects and repairs neurons, improved the degenerative diseases of the central nervous system symptoms, prevent PD (49, 50). In this study we also forecasted the lactose as GLB1 targeted drug, which was converted to galactose in the small intestine. Previous studies have found that galactose, lactose can induce brain aging and increase the risk of PD by causing mitochondrial dysfunction, increasing oxidative stress, inflammation, and apoptosis (51). These evidence provided clues for drug treatment and prevention of PD.

Moreover, in this study, the samples used in the GSE100054 dataset are peripheral blood mononuclear cells, while the samples in the GSE99039 dataset are whole blood. During the expression analysis, it was found that the expression trends of the five biomarkers were consistent in the two datasets. We speculate that this might be because experimental methods such as RNA extraction methods and sequencing depth have limited our ability to accurately detect the subtle differences in biomarker expression between peripheral blood and whole blood samples. However, this consistency may also reflect the stability and universality of the biomarkers in different blood components, which to some extent supports the reliability of these genes as potential biomarkers. Finally, to further verify the results of this study, we conducted RT—qPCR analysis based on clinical samples. The results showed that the expression levels of GLB1, ASAH1 and PSAP in the Parkinson’s disease (PD) group were significantly higher than those in the control group, which further supports our finding that these five small molecule RNA genes (SMRGs) may be biomarkers for Parkinson’s disease. Perhaps we can diagnose Parkinson’s disease by detecting the expression levels of these five SMRGs.

## 5 Conclusion

In conclusion, our study screened SMRGs, analyzed their functions, signaling pathways, immune mechanisms, and explored their molecular regulatory mechanisms and related drugs. Five SMRGs that may be biomarkers of PD were revealed for the first time, further understanding the driving mechanism of PD. These findings were helpful for the early diagnosis of PD, provided targets for the treatment of PD, and offered new research ideas for the study and treatment of the disease. Of course, there were some shortcomings in our study. For example, we have collected multiple datasets in public databases, but the sample size in this study needed to be expanded. We did not further address the relationship between genes involved in sphingolipid metabolism and clinical symptoms of PD.

Moreover, relying solely on blood samples may not accurately reflect the pathophysiological processes of the disease in the central nervous system, and the quantity and heterogeneity of the samples may lead to differences in expression levels. In the future, we will further increase the sample size, obtain more clinical data, conduct larger scale clinical studies, and carry out multi center collaborations to improve statistical effectiveness. This will provide a solid foundation for further investigation of disease mechanisms, optimization of diagnostic methods, and development of more effective treatment strategies.

## Data Availability

The datasets analyzed during the current study are available in the GEO repository with access numbers GSE100054 and GSE99039, https://www.ncbi.nlm.nih.gov/geo/.
