# Supplementary figures and images for: Exploring sphingolipid metabolism-related biomarkers for Parkinson’s disease: a transcriptomic analysis
Source: Front Neurol. 2025 Jun 4;16:1548322. doi: 10.3389/fneur.2025.1548322 (PMC12173885; doi:10.3389/fneur.2025.1548322)

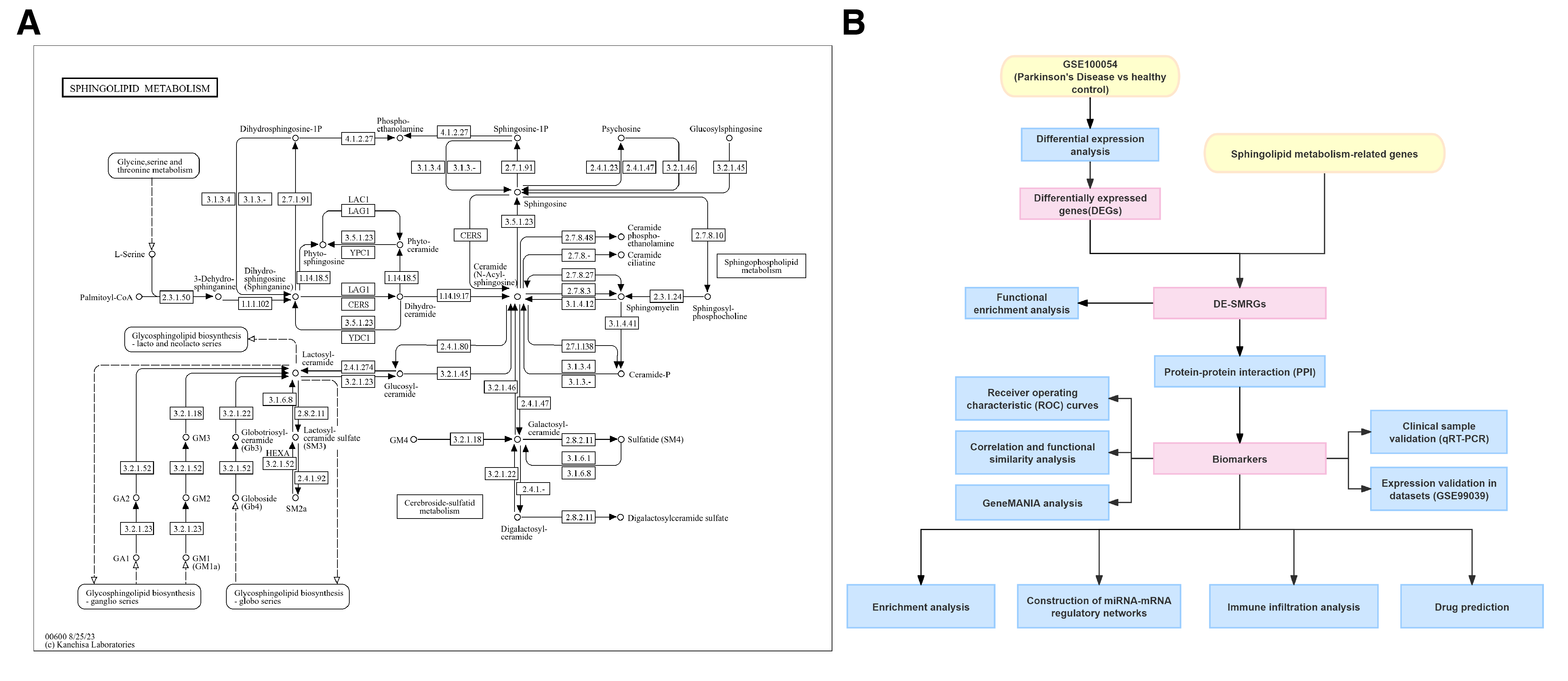

Supplement: Supplementary file 2 [file Image_1.TIF]
